# The Impact of Sociodemographic, Nutritional, and Health Factors on the Incidence and Complications of COVID-19 in Egypt: A Cross-Sectional Study

**DOI:** 10.3390/v14030448

**Published:** 2022-02-22

**Authors:** Mona Schaalan, Ahmed E. Abou Warda, Samir M. Osman, Shaimaa Fathy, Rania M. Sarhan, Marian S. Boshra, Neven Sarhan, Sayed Gaber, Ahmed Mahmoud Abdelhaleem Ali

**Affiliations:** 1 Clinical Pharmacy Department, Faculty of Pharmacy, Misr International University, Cairo 11828, Egypt; mona.schaalan@miuegypt.edu.eg (M.S.); shaimaa.fathy@miuegypt.edu.eg (S.F.); sarhanneven@gmail.com (N.S.); 2 Clinical and Translational Research Unit, Clinical Pharmacy Department, Misr International University, Cairo 11828, Egypt; 3 Clinical Pharmacy Department, Faculty of Pharmacy, October 6 University, P.O. Box 12585, Giza 12585, Egypt; ahmed.essam@o6u.edu.eg; 4 Pharmacognosy Department, Faculty of Pharmacy, October 6 University, P.O. Box 12585, Giza 12585, Egypt; samirothman1965@yahoo.co.uk; 5 Clinical Pharmacy Department, Faculty of Pharmacy, Beni-Suef University, P.O. Box 62514, Beni-Suef 62511, Egypt; raniamohammad87@yahoo.com (R.M.S.); mariansobhy31@yahoo.com (M.S.B.); 6 Critical Care Medicine, Faculty of Medicine, Cairo University, Giza 12211, Egypt; drsayedgaber@hotmail.com; 7 Department of Pharmaceutics and Industrial Pharmacy, College of Pharmacy, Taif University, P.O. Box 11099, Taif P Code 21944, Saudi Arabia

**Keywords:** COVID-19, Egypt, nutritional attitude, precautionary measures and practices, post-COVID-19 symptoms

## Abstract

This study was intended to explore sociodemographic, nutritional, and health-related factors on the incidence of COVID-19 infection within the Egyptian population by assessing the frequency and determinants of post-COVID-19 symptoms and complications. A cross-sectional study using a structured survey on 15,166 participants was adopted. The results revealed common symptoms including fever (79.1%), cough (74.5%), anosmia& ageusia (68.4%), and dyspnea (66.9%). The patients were nonsmokers (83.9%), while 9.7% were mild smokers. The percentage of infected patients with comorbidities versus those without comorbidities were 29%, 71%, respectively. The highest incidence of infection was in those patients with hypertension (14.8%) and diabetes (10.9%), especially females with age >50 years and obesity (BMI; 30–39.9). The highest risks were observed for anticoagulants in the age above 50 years, morbid obesity, presence of comorbidities, and being a healthcare worker. The predictors of clot risk were in the age above 50 years, non-educated, and eating meat and eggs. Nonetheless, the highest risk of using antidepressants was in patients >50 years and those who traveled abroad. These findings and similarities within the surrounding region, the Middle East, North Africa, and South Europe, indicate the possibility of sharing the same viral strain and characteristics that may predict a similar vaccine efficacy and response.

## 1. Introduction

Since December 2019, the whole world has been encountering an unprecedented outbreak of COVID-19, which has originated in Wuhan, China [1,2]. In February 2020, the first COVID-19 case was witnessed in Egypt and Africa. Shortly afterward, in March 2020, the pandemic spread globally, affecting 118,000 people in 114 countries, causing 4291 deaths worldwide [3]. In January 2022, the total worldwide confirmed cases reached more than 321 million, deaths over 5.5 million and total given vaccine doses over 9.5 billion as reported by the John Hopkins University center for systems science and engineering. Resembling the neighboring countries, the Ministry of Health in Egypt declared a state of health emergency and enforced precautionary public health actions that included the mandatory use of masks, social distancing, and quarantine for suspected patients [4]. 

As of February 2021, the number of confirmed cases of COVID-19 in Egypt has ascended to 165,525 and 9460 deaths as per the reports acknowledged by the Ministry of Health and Population. In comparison to the global scale, these relatively low figures may be rationalized by the low testing capacity in the authorized laboratories besides the prevalence of mild to moderate cases that are home managed [5]. Moreover, the social and economic impact should be considered along with other long-standing health issues such as comorbidities and chronic diseases such as hypertension, diabetes mellitus, other cardiovascular diseases, and annual outbreaks of influenza strains [6]. Egypt has already started its vaccination campaign in mid-January 2021, prioritizing elderly patients, those with comorbidities, and healthcare workers. However, since the third wave had already started and was peaking the majority of Egyptians not yet vaccinated, the hitherto anticipated protection was not to be expected.

Although positive PCR remains the gold standard for diagnosis, seroconversion does not mean complete recovery. Hence, monitoring of these subjects for prolonged periods is recommended to evaluate potential post-COVID-19 manifestations and early intervention with the critical warning signs. Continuous counseling is also warranted to assure adherence to the medications that could have a beneficial effect on patients’ outcomes [7].

A large cohort in Egypt experienced post COVID-19 symptoms as well as complications [8]. However, limited studies addressed the factors associated with the pre- as well as post-COVID-19 manifestations. Considering the scarcity of the data about post-COVID-19 manifestations in the literature, many studies worldwide are interested in exploring the factors associated with post-COVID-19 sequelae. 

In this study, our goal was to explore the sociodemographic, nutritional factors as well as health risks on the prevalence of COVID-19 disease and its sequelae within the Egyptian population. Moreover, we aim to address the susceptible social cohorts and their risk factors and to effectively contribute to the design of therapeutic strategies as well as preventive national health approaches of the vaccination campaigns in Egypt.

## 2. Materials and Methods

### 2.1. Study Design and Population

The design of the current study is a cross-sectional survey using a convenient sampling technique. A structured questionnaire was designed in Arabic, the native language in Egypt, and the participation in this study was anonymous and voluntary. The structure of the survey covered the association between the socio-demographic, educational, and nutritional characteristics on the incidence of post-COVID-19 symptoms and complications. Therefore, the survey was categorized into COVID-19 risk factors and disease outcomes. The addressed risk factors were sociodemographic, health risks/comorbid conditions, and nutritional habits, while the outcomes included COVID-19 infection, symptomatology, diagnostic tools applied, isolation incidence, precautionary measures practices as well as post-COVID-19 symptoms and complications.

The “sociodemographic variables” section included items surveying age, sex, height, weight, BMI, marital status, residency, and educational level. The “health risks” section comprised five items including work habits, smoking, comorbid conditions, and chronic medications. The nutritional and dietary behaviors include healthy food, drinks, vitamin supplements, and other food habits. 

### 2.2. Validation and Pilot Study

To confirm the validity and reliability of the questionnaire, a preliminary phase was conducted before its distribution. An Egyptian epidemiologist and a clinical research statistician volunteered to assess the degree of relevance of the survey items to correctly monitor the factors impacting the prevalence of COVID-19 among the Egyptian public.

### 2.3. Data Collection

This study started the enrollment of eligible participants in October 2020 and was ended in January 2021. During that period, the prevalent strain was the D 614G mutant strain, called Wuhan alpha strain. The majority of surveys were distributed to literate participants via an online Google Form (96.1%), and the participants were invited to complete and submit the form. Several surveys were also collected through personal interviews with illiterate and old-aged participants (3.9%); all were conducted via convenient sampling. The survey was distributed to cover the main regions in Egypt, i.e., Central (Great Cairo), Northern Egypt (Alexandria), and Upper Egypt. Cairo is the capital of Egypt and the largest city in terms of population. North Egypt contains cities on the Mediterranean Sea, such as Alexandria, which is the second biggest city in Egypt. Upper Egypt describes the southern cities in Egypt, which are warmer than the northern cities.

### 2.4. Statistical Analysis of the Data

The first test applied was the Kolmogorov-Smirnov test to assess the normality of the data analyses. Quantitative data were described using range, median and interquartile range (IQR) for non-normally distributed data or mean and standard deviation for normally distributed data. Qualitative data were described using numbers and percentages. The chi-square test was used for categorical variables to compare between different groups, the Independent Student t-test was used for normally distributed quantitative variables and Mann-Whitney U test was used for non-normally distributed quantitative variables to compare between two studied groups. Multiple logistic regression analysis found that most independent risk factors present at COVID-19 were associated with a higher incidence of outcome: post-COVID-19 symptoms, depression, clot formation, and use of anticoagulants.

Variables were considered covariates if they showed a significant association in the univariate analysis. The significance of the obtained results was judged at *p* < 0.05. The software used for statistical analysis is IBM SPSS software package version 20.0. (IBM Corp.: Armonk, NY, USA).

## 3. Results

### 3.1. Sociodemographic Data of All Participants 

Fifteen thousand and one hundred sixty-six persons (15,166) from 23 governorates completed the considered survey. The percentage of people who were infected with COVID-19 was 5103 (33.6%), while 10,063 (66.35%) were non-infected. Table 1 displays the socio-demographic characteristics of the studied participants. The ratio of male/female in the non-infected population was 43.7/56.3%, whereas in the infected cohort it was 47.2/52.8%. Around 10,109 (66.6%) of the participants were aged 18 to less than 30 years. Of these, around 2152 (42.17%) were infected.

Those people aged between 30 to 50 years were 3187 (21.01%) in number, whereas only 12.33% of them were aged 50 years and above. The infected/non-infected patients aged between 30–50 totaled 1631/1556 (31.96%/15.46%), while the ratio in the cohort above 50 years old totaled 1320/550 (25.87%/5.47%).

As for the height and weight of the participants (Table 1), represented as their body mass index (BMI), the majority of participants (6669;44%) had normal BMI (18.5–24.99), 37.2% (5638) were overweight, and 2209 (14.6%) were obese, while 316 (2.1%) were underweight and 334 (2.2%) severely obese.

The distribution of the participants all over Egypt was as follows: 7198 (47.5%) in the northern cities of Egypt, followed by 4810 in Greater Cairo (31.7%), while Upper Egypt had 3158 (20.8%). The percentage of infected patients according to their residence was as follows: 2527 in Northern Egypt (49.5%), 1573 (30.8%) in Cairo, and 1003 (19.7%) in Upper Egypt.

As regards marital status, most of the participants were single (9499, 61.5%), a35.4% were married (5366), 171 (1.1%) were engaged, and 300 (2%) were widows. The infected patients in these categories were as follows: 2892 (56.5%) among married people, 1928 (37.8%) single, 215 (4.2%) widows, and 68 (1.3%) engaged.

As for the educational level, the majority of participants had a technical education (6168, 40.7%), there were 5502 (36.3%) students, there were 1698 (11.2%) bachelor’s degree holders, and there were 1153 (7.6%) postgraduate studies holders, while 596 (3.9%) were non-educated. The infected patients showed the highest number among the technical education holders (2250, 44.1%), followed by 988 (19.4%) in the student’s section, 952 (18.7%) bachelor’s degree holders, 475 (9.3%) in postgraduate studies, and 414 (8.1%) in non-educated cohorts.

Table 2 presents the symptomatology, diagnostic measures, and place of isolation of COVID-19 patients. The survey revealed that the common symptomatology encompassed the presence of fever (79.1%), cough (74.5%), loss of sense of smell and taste (68.4%), dyspnea (66.9%), diarrhea (39.8%), while asymptomatic patients totaled only 0.2% (Table 2).

Concerning the diagnostic measure used, the most reliable and economic measures were as follows: clinical examination by a physician (3041, 59.6%), followed by hematological laboratory investigations, including CRP, CBC, ferritin, and D-Dimer (2855, 55.9%) and CT (2358, 46.2%). Conversely, the performance of a PCR test was only 1182 (23.2%).

The place of isolation and treatment reflected the severity of the infected cases. While most of the infected cases were mild to moderate and were home isolated and treated (4246, 83.2%), 16.8% were hospital admitted and isolated (857, 16.8%).

### 3.2. Assessment of the Impact of Social, Nutritional Risk Factors, and Comorbid Health Status on the Incidence of COVID-19 Infection

Table 3 presents the social risk factors affecting the incidence of infection of COVID-19 in terms of occupation, having traveled abroad, and smoking habits. Concerning the occupation of the participants in this study, 6013 (39.6%) were students, 4560 (30.1%) were non-healthcare professionals, 2456 (16.2%) were healthcare professionals, while 2137 (14.1%) were jobless (Table 3). The highest-infected cohort were the non-healthcare professionals (2170, 42.5%), while 1064 (20.9%) were students, 988 (19.4%) were jobless, and 881 (17.3%) were healthcare professionals.

The question about having traveled abroad revealed that 13,581 (89.5%) did not travel abroad, while 1585 (10.5%) traveled outside Egypt. The number of infected patients who did not travel abroad was 4574 (89.6%), while the number of those who did travel totaled 529 (10.4%).

The number of nonsmokers among the participants were 13,165 (86.8%), while those who smoked from 1–10 cigarettes per day totaled 1124 (7.4%) and those who smoked more than 20 cigarettes totaled 227 (1.5%). The infected patients were predominately nonsmokers (4279, 83.9%), while those infected in the smokers of 1–10 cigarettes totaled 493 (9.7%). Those who smoked from 11–20 cigarettes totaled 251 (4.9%), and those who smoked more than 20 cigarettes totaled 80 (1.6%).

Looking at the nutritional habits of the tested cohorts (Table 3), the analysis revealed that 9094 (60%) did eat healthy food. Of these, 59.6% (3040) were COVID-19 infected. On the other hand, those who consumed fast food were 5570 in number. Of these, 37.2% (1900) were infected, while, of the 502 (3.3%) who were vegetarian, only 3.2 (163) were infected with COVID-19. Interestingly, no significant difference was revealed between the infected vs non-infected cohort.

The sort of food eaten by the participants was also assessed. The number of those who ate legumes and beans was 9151 (60.3%), of whom 3060 (60%) were infected, while the number of those who are vegetables and fruits was 11,065 (73%), of whom 3760 (73.7%) were infected. The participants who ate meat and eggs totaled 12,053 (79.5%), of whom 4065 (79.7%) were infected. Interestingly, no significant difference was revealed between the infected vs non-infected cohort.

Moreover, the prevalence of COVID-19 infection among patients with comorbidities is depicted in Table 3. The number of participants who suffered from chronic comorbid diseases was 2732, constituting 18% of the total participants (15,166), while those without comorbid diseases were 12,434 (82.0%). The infected patients with comorbidities totaled 1479 (29%), while 3624 (71%) were infected without comorbid diseases. The type of chronic disease and their percentage among infected and non-infected cohorts are illustrated in Table 3. The highest rate of infection in the chronic diseases was in those with hypertension (756, 14.8%), followed by diabetes mellitus (558,10.9%), chest diseases (254, 5%), cardiac diseases (166, 3.3%), autoimmune diseases (108, 2.1%), hepatic diseases (47, 0.9%), cancer (13, 0.3%), and renal failure (23, 0.5%).

The impact of the applied precautionary measures on the rate of infection with COVID-19 is presented in Table 4. The number of participants using multivitamins was 3281 (21.6%), of whom 1098 (21.5%) were infected, while 2183 (21.7%) were not infected, which reveals a nonsignificant difference between the infected and non-infected cohort taking multivitamins.

In contrast, 11,885 participants (78.4%) did not use multivitamins, of whom 4005 (78.5%) were infected, while 7880 (78.3%) were not infected. No significant difference was observed between infected versus non-infected cohorts using multivitamins.

Of the participants receiving the annual influenza vaccine (8666; 57.1%), the infected patients totaled 4020 (78.8%), those protected 4646 (46.2%). On the other hand, those not previously vaccinated who caught COVID-19 infection were 1083 (21.2%) in number, while those protected were 5417 (53.8%). The latter reflected a significant difference between both cohorts.

The implementation of the precautionary measures was highest in wearing the face mask (12,813, 84.5%), followed by using alcohol for disinfection (9069, 59.8%), wearing a face shield (2263, 14.9%), and those who did not follow any precautionary measures (1617, 10.7%). The impact of the precautionary measures applied in the community on the rate of infection, compared to those not infected, revealed a significant difference only with those wearing the face mask (*p* = 0.006). However, the other measures did not reveal a significant difference: alcohol for disinfection (*p* = 0.066) and wearing a face shield (*p* = 0.83).

Questioning if the participants had any contact with COVID-19 infected patients revealed that 6500 participants did not interact with any infected patients, of whom 1083 (21.25%) were infected and 5417 (53.8%) were not. Of those who were in contact with COVID-19 infected patients, 4020 (78.8%) caught the infection, while 4646 (46.2%) did not.

Table 5 focuses on the incidence of clots and depression among infected patients as well as the post-COVID-19 symptoms and complications. The incidence of clots was significantly higher in infected patients (192; 3.8%) compared to those non-infected (58; 0.6%). Moreover, the incidence of depression and usage of antidepressants was higher in infected patients (336; 6.6%) compared to those non-infected (336; 3.3%). Those who did not experience any complications despite their infection were 4459 (87.4%) in number, while non-infected participants totaled 9415 (93.6%).

The number of infected patients who experienced post COVID-19 symptoms was 1093 (21.4%), while those who did not were 4010 (78.6%). Moreover, those who received anticoagulants were 2015 (39.5%) in number, while those who did not totaled 3088 (60.5%).

### 3.3. Post COVID-19 Symptoms

The current survey is the first attempt to investigate the multivariate logistic regression to determine the correlation between the sociodemographic characteristics, comorbidities, and other factors with predictive post-COVID-19 sequelae. The significant predictive covariates associated with post-COVID-19 symptoms, the use of antidepressants, the incidence of clots, and the use of anticoagulants are illustrated in Table 6.

Concerning the risk of post-COVID-19 symptoms, females had a 1.6 times higher risk of experiencing post-COVID-19 symptoms compared to males (CI; 1.44–1.96). Concerning the impact of the BMI, the obese patients (BMI; 30–39.99) exhibited a 1.5 times higher risk of post-COVID-19 symptoms (CI; 1.16–1.96) than the lean patients.

As for the risk of using anticoagulants, the above 50 showed a 1.72 higher risk than the reference group. Moreover, the odds of using anticoagulants presented a linear correlation with the increase in BMI, with the highest risk in severely obese patients (BMI ≥ 40) (OR; 3.420 and CI; 2.39–4.90), followed by Low BMI (<18.5) with OR 2.048 and CI: (1.14–3.68), obese patients (30–39.99) with OR 1.94, (CI; 1.63–2.31), and the lowest in overweight patients (25–29.99) with OR 1.42 (CI; 1.23–1.64).

The use of anticoagulants also led to a higher risk (OR; 1.387 and CI; 1.19–1.62) for healthcare professionals than for other non-healthcare workers.

As for the impact of comorbidities on the risk of requiring anticoagulants, the risk was two times higher in patients with comorbid diseases, compared to those without comorbidities.

Apropos the incidence of clots, the risk was 3.4 times higher in the aged cohort above 50 years (CI; 1.81–6.65), while the risk in non-educated cohorts was 1.67 times higher (CI; 1.11–2.52).

Patients who traveled outside the country were at higher risk of depression than those who did not (OR; 1.622, CI 1.17–2.24).

## 4. Discussion

This study sheds light on the epidemiological, nutritional, and health-related facets of COVID-19 infected patients during the current pandemic as well as the associated determinants and predictors of post-COVID-19 complications in Egypt.

We investigated 15,166 COVID-19-exposed participants, of whom 5103 (33.64%) were infected with active diseases with typical symptoms. Among the infected cohort, females constituted 52.8%, which reflects the equal exposure of working females in the community. The highest infection incidence was in the age range of 18–30 years (42.2%), followed by the 30–50-year-old cohort (21.01%), whereas only 12.33% were aged 50 years and above. Despite the alignment of our results with those reported globally, some manifestations require further follow-up. A plethora of studies from other countries showed higher median age [9,10] and mean age [11] for SARS-CoV-2 infection. We rationalized our observations in light of the socio-economic status in Egypt, where the 18–40-year-old cohort is the predominantly young and middle-aged working population, who are leaving their homes for work, and hence are more exposed to the infection [12].

As for the body mass index (BMI), most participants had normal BMI (18.5–24.99), followed by overweight patients (44%, 37.2%; respectively), which reflects the contribution of obesity to the incidence of infection.

Obesity is reported to have an increased risk for severe diseases, hospital admission, and stay, as well as high mortality in the current COVID-19 pandemic disease [13]. Recent reports have revealed that obesity causes a waning of the immune system and increases thereby the vulnerability to infectious diseases. Obesity with excess adiposity is evidenced to induce interruption of the equilibrium between pro-inflammatory and anti-inflammatory immune cells, with a predominance of the former [14]. This disturbance induces a chronic low-grade inflammatory milieu, which is exaggerated by an intense inflammatory storm arising from the COVID-19 infection itself, resulting in more severe disease pathology and worse sequelae [15].

Looking at the educational level of the infected patients, the highest number was among the technical education holders, followed by the students, constituting 44.1% and 19.4%, respectively. This could be explained by the working capacity of each cohort, i.e., the technical workers are the highly recruited cohort, working in the industry, factories, and private markets and consequently highly exposed to the infection. Inherently, our observation identified only 0.2% who presented no symptoms. It is known that asymptomatic COVID-19 manifestation is more common among younger people [16]. The number of asymptomatic patients in previous studies was higher, as Manann et al. (2021) reported 10.9% asymptomatic cases, which is following the findings in South Korea and China [9,17]. The latter finding may be due to genetic diversity or new viral mutations responsible for asymptomatic cases [18,19].

Concerning the diagnostic measures used for COVID-19, our study delineates that the majority of suspected patients were diagnosed by clinical examination (59.6%), which is the most reliable and economic measure performed, followed by hematological and biochemical laboratory assessments, including CRP, CBC, ferritin, and D-Dimer (55.9%). Further confirmation was performed by CT scan (46.2%) to determine any lung inflammation or ground-glass opacities; conversely, the performance of a PCR test was conducted by only 1182 participants (23.2%) due to its relatively high cost and the inability of the lower and possibly the middle social classes to afford its fee.

Regarding the occupation of the infected cohort, the highest infection was reported in the non-healthcare professionals (NHCP), followed by students (42%, 20.9%; respectively). Although the healthcare professionals (HCP) constituted 17.3%, they are at increased infection burden, being exposed to a high viral load every day.

In the current study, the majority of participants (78.8%) had close contact with confirmed cases; these data suggest that community transmission is possibly common, hence social distancing, and that all precautionary procedures should be fully applied to impede further deterioration [20]. Moreover, our study revealed that among the infected patients, 529 (10.4%) did travel outside Egypt, which may be the reason for infection, while 4574 (89.6%) did not travel abroad.

Concerning the incidence of COVID-19 infection in smokers versus nonsmokers, in the current study, the majority of infected patients were predominately nonsmokers (83.9%), while those infected in the smoking cohort were ranked according to the intensity of smoking. The percentage of infected patients who smoke from 1–10 cigarettes was 9.7%, followed by those who smoke from 11–20 cigarettes (4.9%) and more than 20 cigarettes constituted 1.6%. In the latter results, two phenomena need to be addressed and highlighted. First, the majority of infected patients are nonsmokers, contradicting the reported hypothesis in the literature that smoking predisposes smokers to respiratory infections. The second interesting phenomenon is that the lower the smoking intensity, the higher the incidence of infection. This inverse association between smoking and the risk of infection has been lately highlighted in several studies as a focus of debate on the reported controversial findings and respective pieces of evidence.

The rational mechanisms of the suggested protective effect of smoking comprise an anti-inflammatory effect of nicotine, thereby reducing the cytokine storm-induced weakening of the immune response. Moreover, the nicotine-induced inhibition of SARS-CoV-2 replication in the pulmonary cells via stimulation of nitric oxide cannot be excluded [21].

Another explanation was provided by Tanimoto et al., (2021), who reported that cigarette smoke extract (CSE) treatments were, surprisingly, found to suppress the expression of ACE2 in HepG2 cells [22]. Interestingly, the effect of smoking on the Antibody titer after vaccination is inverted. In the study of Nomura et al., (2021), the Brinkman index and the number of cigarettes per day decreased the Ab titers. This indicates that smoking itself is considered a risk factor for low Ab titers, rather than the duration of smoking or number of cigarettes per day. Furthermore, smoking cessation is anticipated to significantly increase Ab titers because they were significantly lower in current smokers than in ex-smokers [23]. Many risk factors for COVID-19 infection have been studied in the current study to investigate their association with the incidence of infection.

Looking at the nutritional habits of the tested cohorts, the current survey analysis revealed that 60% of the infected cohort consumed healthy food, 37.2% consumed fast food, and only 3.3% were vegetarian.

In the present study, the influence of the applied precautionary measures on the incidence of infection with COVID-19 revealed that the annual vaccination did not offer any protective advantage. Additionally, participants using multivitamins showed no noteworthy protection against the infection versus those who were non-infected. This finding is in line with the results of a meta-analysis where vitamin C (1 g/day) supplementation did not prove any therapeutic effect on upper respiratory tract infections in a large meta-analysis of 11,306 participants [24]. Intriguingly, other controversial findings were reported concerning the protective role of vitamin C supplementation in COVID-19 infection. In another small study on 17 COVID-19 patients, vitamin C (1 g, IV) succeeded in decreasing the extent of inflammation, including ferritin and D-dimer, thereby improving the oxygen requirements and hemodynamic parameters and reducing COVID-19-induced lung inflammation and injury [25].

The implementation of COVID-19 precautionary measures, in the current study, was highest in those wearing the face mask (84.5%), followed by those using alcohol for disinfection (59.8%). The impact of the precautionary measures applied in the community on the rate of infection, compared to those not infected, reflected a significant difference only with those wearing the face mask, and using alcohol did not show much significance [20]. These findings reflect that more awareness of the precautionary measures of wearing masks and social distancing is still needed in the community as well as in numerous governmental and private settings.

Concerning the comorbidities among the study participants, 2732 from the total of 15,166 study participants (18%) had comorbid diseases, of whom 1479/5103 (29%) were infected. Hypertension was found to be the most prominent comorbidity (14.8%), followed by diabetes mellitus (10.9%), findings that were also revealed in similar studies performed in the UK and China [12,15]. Other remarkable comorbidities were respiratory, chest (5%), and cardiovascular (3.3%) diseases, which aligned with the results of high prevalence obtained from our study as well as tendencies in other countries [10,11,15]. Other diseases include autoimmune diseases (108, 2.1%), hepatic diseases (47, 0.9%), cancer (13, 0.3%), and renal failure (23, 0.5%).

Some beneficial lessons were learned from the previous severe acute respiratory syndrome (SARS) attack in 2003, and many common symptoms were reported between post-SARS recovery manifestations and post-COVID-19 symptoms, such as fatigue, myalgia, depression, and weakness [26].

Reviewing recent studies, approximately 87% were still suffering at least one symptom 1–2 months after recovery from COVID-19 infection. The wide range of symptoms included respiratory symptoms (e.g., breathing difficulty, chest pain, and cough), neurological symptoms (e.g., headache, lethargy, palpitations/tachycardia, sleeping troubles, joint ache), and neuropsychiatric symptoms such as depression [27]. The latter post-COVID-19 symptoms are considered among the most common neurological manifestations that could be classified into three categories: skeletal muscle symptoms (e.g., myalgia and rhabdomyolysis), peripheral nervous system symptoms (e.g., taste and smell impairment and nerve pain), and central nervous system symptoms (e.g., dizziness, headache, confusion consciousness, cerebrovascular diseases, and seizures), beyond the frequently witnessed neuro-psychiatric symptoms, such as depression [28].

The neurological manifestation of post-COVID-19 could be classified as mild or critical, with most of the investigated manifestations being mild reversible symptoms such as fatigue, headache, joint, and muscle pain. On the other hand, the critical manifestations are those affecting organ functions. The confirmatory measures used for the diagnosis of severe cases were laboratory investigations and imaging diagnostic techniques such as CT scans for the diagnosis of pulmonary fibrosis [29]. A recent Chinese study found that confusion and headache are the most observed among the presented neurological complications. Intriguingly, anosmia and ageusia are symptoms that arise before pulmonary symptoms, suggesting early neurological sequelae [2].

In our study, the number of infected patients who experienced post COVID-19 symptoms was 21.4%, while those who did not were 78.6%. The multivariate logistic regression testing the association of risk factors of COVID-19 patients with a higher incidence of post-COVID-19 symptoms revealed that the female sex had a 1.6 times higher risk of post-COVID-19 symptoms (OR; 1.618; CI 1.44–1.96) compared to males. Concerning the impact of BMI, the obese patients (BMI; 30–39.99) were significantly correlated with post-COVID-19 symptoms, with 1.509 higher risks (CI; 1.16–1.96).

Curiously, of the various respiratory post-COVID-19 symptoms, dyspnea was one of the common post-COVID-19 symptoms reported in a previous study of Iqbal et al. (2021) [30]. The presence of dyspnea in post-COVID-19 recovery could be rationalized by the similarity to SARS survivors who experienced respiratory exacerbations due to abnormalities in pulmonary functions, e.g., forced vital capacity, abnormalities in total lung capacity as well as diffusion in lung capacity of carbon monoxide, even at six months post-discharge [31]. The association between persistence of post-recovery symptoms (e.g., fatigue, dyspnea, headache, and time) since recovery from COVID-19 was also previously depicted [32].

Several reports found that the susceptibility of recovered patients towards post-COVID-19 complications was related to having previous chronic comorbidities. Our findings are in alignment with similar studies in other countries [27,33].

Iqbal et al. (2021) [30] reported a significant association between the severity of COVID-19 disease manifestations and post-COVID-19 symptoms. These results match those of Kamal et al. (2020), showing that severe COVID-19-infected Egyptian patients encountered more severe post-recovery manifestations than those with milder disease [34]. These results are also in harmony with the studies performed in Italy, the United Kingdom (UK), and Egypt. In these studies, they followed up patients for several months and reported fatigue as the most prevalent post-COVID-19 manifestation [35]. Furthermore, the incidence of depression and usage of antidepressants was higher in infected patients (336; 6.6%) compared to those non-infected (336; 3.3%). In our study, patients who traveled outside the country were on antidepressants more than those who did not OR 1.622, CI 1.17–2.24, and were also home isolated with OR 0.764, CI; 0.60–0.97. 

Depression has been reported by our participants as one of the reported neurological post-COVID-19 symptoms; others include confusion, poor sleep quality, and anxiety. It was prominent that these CNS manifestations included permanent headache, migraine, anxiety, obsessive-compulsive disorder (OCD) besides depression [35].

The incidence of using anticoagulants among the infected group in our study was 39.5% while those who did not were 60.5%. The number of patients who did not experience any complications despite their infection was 4459 (87.4%), while in non-infected participants it was 9415 (93.6%).

As for the risk of using anticoagulants, the patients >50 years showed a 1.72 higher risk than their reference group (1.38–2.14). Moreover, the odds of using anticoagulants showed a linear correlation with the increase in BMI. The highest risk for using anticoagulants was in severely obese patients (BMI ≥40) with OR; 3.420 (CI; 2.39–4.90), followed by those with low BMI (<18.5) with OR 2.048 (CI: 1.14–3.68), who were obese (30–39.99 with OR 1.94 (CI; 1.63–2.31), while the least risk was in overweight patients (25–29.99) with OR 1.42 (CI; 1.23–1.64).

Remarkably, in our current study, patients with comorbid diseases have double the risk of using anticoagulants compared to those without comorbidities (CI; 1.10–3.76). Several reports shed light on the association between obesity and hypercoagulability state with elevated levels of surrogate prothrombin factors and reduced anti-thrombin levels [36]. Regarding the impact of obesity as an independent variable impacting COVID-19 severity or post COVID-19 syndrome, studies have shown that obesity in the infected patients had no significant impact on the severity score or type of post-COVID-19 symptoms [34].

The use of anticoagulants was also at higher risk for healthcare professionals than other non-healthcare workers. (OR 1.387 and CI; 1.19–1.62).

The current study also addressed the incidence of hematological complications such as clots among infected patients. The incidence of clots was significantly higher in infected patients (192; 3.8%) compared to those non-infected (58; 0.6%).

There is a piece of evolving evidence advocating that COVID-19-induced pulmonary thrombo-embolism is considered an under-recognized complication, for which chronic pulmonary hypertension is a major risk [37]. Furthermore, previous studies have shown that acute lung injury is associated with pulmonary fibrosis restrictive functional pattern and hence a worse quality of life [38].

For the incidence of clots, the risk is 3.4 times higher in the cohort aged above 50 years (CI; 1.81–6.65), and in non-educated cohorts 1.67 times higher (CI; 1.11–2.52).

The incidence of clots was experienced more in non-educated patients than in those who were educated (OR 1.674, CI; 1.11–2.52). Finally, to the best of the authors’ knowledge, the current study is the most inclusive study conducted in Egypt to have assessed COVID-19 patients for sociodemographic, nutritional as well as clinical aspects of the disease post-COVID-19 sequelae.

The results obtained from our cross-sectional survey align with similar global COVID-19 manifestations and complications in the Middle East region as well as South European countries. This similarity in disease characteristics indicates that the same viral strain is being shared in these countries; hence, a similar vaccine efficacy can be predicted. Nevertheless, further studies about the characteristics of different COVID-19 strains, such as omicron strains, need to be performed in the future.

## 5. Conclusions

Our data have corroborated similar global manifestations and raised some important reflections on COVID-19 incidence, propagation, and complications in Egypt. However, these results are called into question by some limitations. First, the addressed patients’ sample of post-COVID-19 cases cannot represent all post-COVID-19 patients in Egypt. Secondly, considering our inability to lead personal face-to-face interviews, random selection bias may be the case in many samples. Finally, a longer follow-up period is warranted to better understand the progression of symptoms post COVID-19. Further studies and research are recommended to better illustrate persistent post-COVID-19 symptoms and their correlation with pre-COVID-19 conditions in various community and clinical settings.

## Figures and Tables

**Table 1:** The demographic data and the incidence of COVID-19 infection.

	Total (*n* = 15,166)		Did You Get an Infection with COVID-19?				Test of Sig.	*p*-Value
			No (*n* = 10,063) 66.35%		Yes (*n* = 5103) 33.64%			
	No.	%	No.	%	No.	%		
**Sex**								
**Male**	6808	44.9	4401	43.7	2407	47.2	χ^2^ = 16.139 *	<0.001 *
**Female**	8358	55.1	5662	56.3	2696	52.8		
**Age (years)**								
**18-<30**	10,109	66.66	7957	79.07	2152	42.17	χ^2^ = 2273.287 * t = 45.025 *	<0.001 * <0.001 *
**30–50**	3187	21.01	1556	15.46	1631	31.96		
**≥50**	1870	12.33	550	5.47	1320	25.87		
**Mean ± SD.**	30.32 ± 13.20		26.68 ± 9.93		37.51 ± 15.66			
**Height (cm)**								
**Median, (IQR)**	168.0 (160.0–175.0)		167.0 (160.0–175.0)		168.0 (161.0–175.0)		U = 3.716 *	<0.001 *
**Weight (kg)**								
**Median, (IQR)**	72.0 (63.0–83.0)		70.0 (60.0–80.0)		77.0 (68.0–86.0		U = 22.901 *	<0.001 *
**BMI**								
**Low (<18.5)**	316	2.1	267	2.7	49	1.0	χ^2^ = 553.310 *	<0.001 *
**Normal (18.5–24.99)**	6669	44.0	5025	49.9	1644	32.2		
**Overweight (25–29.99)**	5638	37.2	3393	33.7	2245	44.0		
**Obese (30–39.99)**	2209	14.6	1193	11.9	1016	19.9		
**Severely obese (≥40)**	334	2.2	185	1.8	149	2.9		
**Residency**								
**Upper Egypt**	3158	20.8	2155	21.4	1003	19.7	χ^2^ = 13.827 *	0.001 *
**Northern Egypt**	7198	47.5	4671	46.4	2527	49.5		
**Great Cairo**	4810	31.7	3237	32.2	1573	30.8		
**Marital status**								
**Single**	9329	61.5	7401	73.5	1928	37.8	χ^2^ = 1886.503 *	<0.001 *
**Married**	5366	35.4	2474	24.6	2892	56.7		
**Engaged**	171	1.1	103	1.0	68	1.3		
**Widow**	300	2.0	85	0.8	215	4.2		
**Educational level**								
**Non-educated**	596	3.9	182	1.8	414	8.1	χ^2^ =1388.119 *	<0.001 *
**Student**	5502	36.3	4514	44.9	988	19.4		
**Technical education**	6168	40.7	3918	38.9	2250	44.1		
**Bachelor**	1698	11.2	746	7.4	952	18.7		
**Postgraduate studies**	1153	7.6	678	6.7	475	9.3		
**Others**	49	0.3	25	0.2	24	0.5		

SD: Standard deviation χ^2^: Chi-square test; *t*: Student *t*-test; *p*-value: for comparing between the studied categories; (*): Statistically significant at *p* ≤ 0.05. IQR: Interquartile range.

**Table 2:** Symptomatology, diagnostic measures, and place of isolation of COVID-19 patients (*n* = 5103).

Survey Item	No.	%
**Select the Symptoms Associated with the Disease;**		
**Fever**	4035	79.1
**Cough**	3804	74.5
**Loss of sense of smell (anosmia) and loss of sense of taste (ageusia)**	3492	68.4
**Dyspnea**	3414	66.9
**Diarrhea**	2033	39.8
**Without symptoms**	8	0.2
**Other**	322	6.3
**How was it diagnosed?**		
**Clinical examination**	3041	59.6
**Lab. investigation (CRP, CBC, ferritin, D-dimer, etc)**	2855	55.9
**Chest CT**	2358	46.2
**PCR**	1182	23.2
**Where were you isolated?**		
**Home isolation (Mild-Moderate cases)**	4246	83.2
**Hospital (severe cases)**	857	16.8

**Table 3:** Impact of social, nutritional risk factors, and comorbid health status on the incidence of COVID-19 infection.

	Total (*n* = 15,166)		Did You Get an Infection with COVID-19				χ^2^	*p*
			No (*n* = 10,063)		Yes (*n* = 5103)			
	No.	%	No.	%	No.	%		
**Occupation**								
**Jobless**	2137	14.1	1149	11.4	988	19.4	1239.35 *	<0.001 *
**Student**	6013	39.6	4949	49.2	1064	20.9		
**Non-healthcare professional**	4560	30.1	2390	23.8	2170	42.5		
**Healthcare professional**	2456	16.2	1575	15.7	881	17.3		
Previous travel outside the country								
**No**	13,581	89.5	9007	89.5	4574	89.6	0.059	0.808
**Yes**	1585	10.5	1056	10.5	529	10.4		
**Smoking**								
**No**	13,165	86.8	8886	88.3	4279	83.9	67.690 *	<0.001 *
**Irregular or from 1–10 cigarettes**	1124	7.4	631	6.3	493	9.7		
**From 11–20 cigarettes**	650	4.3	399	4.0	251	4.9		
**More than 20 cigarettes**	227	1.5	147	1.5	80	1.6		
What are your food habits?								
**Healthy food**	9094	60.0	6054	60.2	3040	59.6	1.044	0.593
**Fast food**	5570	36.7	3670	36.5	1900	37.2		
**Vegetarian**	502	3.3	339	3.4	163	3.2		
Does your diet contain any of these ingredients?								
**Legumes and beans**	9151	60.3	6091	60.5	3060	60.0	0.450	0.502
**Vegetables and fruits**	11,065	73.0	7305	72.6	3760	73.7	2.037	0.153
**Meat and eggs**	12,053	79.5	7988	79.4	4065	79.7	0.162	0.688
Do you suffer from one of the following diseases?								
**No**	12,434	82.0	8810	87.5	3624	71.0	626.544 *	<0.001 *
**Yes**	2732	18.0	1253	12.5	1479	29.0		
**Hypertension**	1161	7.7	405	4.0	756	14.8	557.655 *	<0.001 *
**Diabetes**	806	5.3	248	2.5	558	10.9	482.758 *	<0.001 *
**Chest disease**	469	3.1	215	2.1	254	5.0	91.189 *	<0.001 *
**Cardiac disease**	250	1.6	84	0.8	166	3.3	122.133 *	<0.001 *
**Autoimmune disease**	240	1.6	132	1.3	108	2.1	14.077 *	<0.001 *
**Hepatic diseases**	69	0.5	22	0.2	47	0.9	36.886 *	<0.001 *
**Cancer**	29	0.2	16	0.2	13	0.3	1.627	0.202
**Renal Failure**	32	0.2	9	0.1	23	0.5	20.990 *	<0.001 *
**Other diseases**	454	2.9	279	2.7	175	3.4	5.070 *	0.024 *

χ^2^: Chi-square test; *: Statistically significant at *p* ≤ 0.05.

**Table 4:** The relation between the applied precautionary measures and the incidence of infection with COVID-19.

	Did You Get an Infection with COVID-19?				Total (*n* = 15,166)		χ^2^	*p*
	No (*n* = 10,063)		Yes (*n* = 5103)					
	No.	%	No.	%	No.	%		
**Are you a regular user of multivitamins?**								
**No**	**7880**	**78.3**	**4005**	**78.5**	**11,885**	**78.4**	0.062	0.803
**Yes**	2183	21.7	1098	21.5	**3281**	21.6		
Are you annually receiving the influenza vaccine?								
**No**	5417	53.8	1083	21.2	**6500**	42.9	1470.083 *	<0.001 *
**Yes**	4646	46.2	4020	78.8	**8666**	57.1		
What are you doing to protect yourself from COVID-19?								
**Do not follow any precautions**	1115	11.1	502	9.8	**1617**	10.7	5.491 *	0.019 *
**Face mask**	8444	83.9	4369	85.6	**12,813**	84.5	7.509 *	0.006 *
**Wear face shield**	1506	15	757	14.8	**2263**	14.9	0.046	0.830
**Use alcohol**	56,965	59.3	3104	60.8	**9069**	59.8	3.386	0.066
**Others**	94	0.9	45	0.9	**139**	0.9	0.102	0.750
Have you had any contact with someone with COVID-19 disease?								
**No**	5417	53.8	1083	21.2	**6500**	42.9	1470.083 *	<0.001 *
**Yes**	4646	46.2	4020	78.8	**8666**	57.1		

χ^2^: Chi-square test; *p*: *p*-value for comparing between the studied categories; (*): Statistically significant at *p* ≤ 0.05.

**Table 5:** The experienced complications among COVID-19 infected patients.

Did You Suffer from Any of the Following Problems since Your Infection	Did You Get an Infection with COVID-19				Total		χ^2^	*p*
	No (*n* = 10,063)		Yes (*n* = 5103)		(*n* = 15,166)			
	No.	%	No.	%	No.	%		
**Clots**	**58**	0.60	192	3.80	250	1.60	212.010 *	<0.001 *
**Depression needs treatment**	336	3.30	336	6.60	672	4.40	84.218 *	<0.001 *
**Not experience anything**	9415	93.60	4459	87.40	13,874	91.50	165.967 *	<0.001 *
**Other**	113	1.10	93	1.80	206	1.40	12.366 *	<0.001 *
**Did you experience post-COVID-19 symptoms?**			**No.**			**%**		
**No**			4010			78.6		
**Yes**			1093			21.4		
**Did you receive anticoagulant**								
**No**			3088			60.5		
**Yes**			2015			39.5		

*p*: *p*-value for comparing the studied categories; (*): Statistically significant at *p* ≤ 0.05.

**Table 6:** Results of multivariate logistic regression between the sociodemographic factors and post COVID-19 symptoms, use of anticoagulant, the incidence of clots, and neuropsychiatric depression symptoms that required antidepressant therapy.

	Post-COVID Symptoms		Use of Anticoagulant		Incidence of Clots		Depression/ Use of Antidepressants	
	*p*	OR (95% C.I)	*p*	OR (95% C.I)	*p*	OR (95% C.I)	*p*	OR (95% C.I)
Sex								
Male ^®“Ref.”^		Ref.						
Female	<0.001 *	1.618 (1.34–1.96)		NA		NA		NA
Age (years)								
18-<30 ^®“Ref.”^		Ref.						
30–50	0.801	0.975 (0.80–1.18)	0.087	1.19 (0.97–1.45)	0.089	1.727 (0.92–3.24)	0.176	0.776 (0.54–1.12)
≥50	0.001 *	0.606 (0.45–0.82)	<0.001 *	1.72 (1.38–2.14)	<0.001 *	3.471 (1.81–6.65)	0.003 *	0.509 (0.33–0.80)
BMI								
Normal (18.5–24.99) ^®“Ref.”^		Ref.						
Low (<18.5)	0.375	1.372 (0.68–2.76)	0.016 *	2.048 (1.14–3.68)		NA		NA
Overweight (25–29.99)	0.320	1.104 (0.91–1.34)	<0.001 *	1.42 (1.23–1.64)		NA		NA
Obese (30–39.99)	0.002 *	1.509 (1.16–1.96)	<0.001 *	1.94 (1.63–2.31)		NA		NA
Severely obese (≥40)	0.428	0.702 (0.29–1.68)	<0.001 *	3.420 (2.39–4.90)		NA		NA
Residency								
Great Cairo ^®“Ref.”^		Ref.						
Upper Egypt	0.005 *	0.696 (0.54–0.90)	0.009 *	0.798 (0.67–0.95)		NA		NA
Northern Egypt	0.045 *	0.824 (0.68–0.100)	0.208	1.089 (0.95–1.24)		NA		NA
Marital status								
Single ^®“Ref.”^		Ref						
Married		NA	0.015 *	1.278 (1.05–1.56)		NA	0.482	0.880 (0.62–1.26)
Engaged		NA	0.739	1.094 (0.64–1.86)		NA	0.001 *	3.023 (1.57–6.53)
Widow		NA	0.427	1.152 (0.81–1.64)		NA	0.281	1.444 (0.74–2.82)
Educational level								
Educated ^®“Ref.”^								
Non-educated		NA		NA	0.013 *	1.67 (1.11–2.52)		NA
Job								
Other ^®“Ref.”^								
Healthcare professional		NA	<0.001 *	1.38 (1.19–1.62)		NA		NA
Previous travel outside the Country								
No ^®“Ref.”^								
Yes		NA		NA			0.004 *	1.622 (1.17–2.24)
Suffering from any of the comorbid diseases		NA	0.024 *	2.031 (1.10–3.76)		NA		NA
Diet: meat and eggs		NA		NA	0.015 *	0.661 (0.47–0.92)		NA

OR: Odds ratio CI: Confidence interval. Any variable that has any significant item in the univariate analysis was included in the multivariate analysis. Only the results of significant variables in the multivariate were shown in bold. *p*: *p*-value for comparing the studied categories; (*): Statistically significant at *p* ≤ 0.05.

## Data Availability

Data available on request from the authors. The data that support the findings of this study are available from the corresponding author upon reasonable request.
